# Smoke-Water Enhances Germination and Seedling Growth of Four Horticultural Crops

**DOI:** 10.3390/plants8040104

**Published:** 2019-04-18

**Authors:** Mohamed A. Elsadek, Eltohamy A. A. Yousef

**Affiliations:** 1 Department of Landscape Architecture, College of Architecture and Urban Planning, Tongji University, Shanghai 200092, China; 2 Department of Horticulture, Faculty of Agriculture, Suez Canal University, Ismailia 41522, Egypt; tohamy_yousef@agr.suez.edu.eg

**Keywords:** germination, horticulture plants, plant derived smoke, α-amylase activity, abscisic acid

## Abstract

The impact of plant-derived smoke as a promoter of seed germination in many crops is well documented. However, very little is known about (1) the appropriate plant species for smoke-water preparation, (2) the effect of smoke-water on the germination and the post-germination parameters in non-fire-prone environments, and (3) the relative importance of dark and light conditions and their possible effects. To fill these gaps in knowledge, we conducted field experiments to evaluate the effect of smoke-water produced from five plant species—white willow, sage, rice straw, rosemary, and lemon eucalyptus—on the germination and seedling growth of cucumber, tomato, scotch marigold, and gladiolus. The seeds and cormels were soaked in smoke-water under light or dark conditions. The results revealed that the smoke-water treatments derived from white willow and lemon eucalyptus enhanced germination, post-germination parameters, and macro element content whilst also contributing to dormancy-breaking. In addition, these smoke-water treatments significantly reduced abscisic acid content and increased α-amylase activity under light conditions; however, the stimulating effects were absent under dark conditions. In conclusion, we provide new evidence that germination and seedling growth in non-fire-prone environments can be enhanced by plant-derived smoke, and that stimulating impacts depend on the plant species used to prepare the smoke-water.

## 1. Introduction

Germination rate is significantly affected by several abiotic and biochemical factors [1]. In developed and developing countries, the residue of the previous crop is often eliminated using fire, and this technique has now been described as “prescribed burning” [2]. Although this practice is convenient and cost-effective, it also has many hazards, including air pollution and killing of beneficial soil microorganisms [3]. Based on prescribed burning, researchers have been able to use smoke resulting from the fire in fire-prone regions to promote seed germination [4]. Subsequently, plant-derived smoke and aqueous extracts obtained from smoke have many applications as tools in the field of agriculture and/or to improve the conservation of threatened or rare species [5,6,7,8]. 

Smoke contains many different compounds, and several attempts have been made to identify these active compounds [9,10]. Four major active compounds with potential agricultural use have been identified and isolated: karrikins (KARs) [11], cyanohydrins [12], butenolides [13], and hydroquinones [14]. KARs and cyanohydrins are heat-stable compounds, and they both exhibit prolonged durability and dissolve in water. They also markedly improve seed germination in many plant species [2]. With regard to horticultural practices, the advantages of smoke can be exploited using three main forms—aerial smoke, smoke-water extracts, and dynamic compounds (KARs). In recent years, studies of plant-derived smoke applications have extended to agricultural and horticultural species, e.g., weed control [2,15,16]. Smoke has also been shown to stimulate seed germination and seedling growth of some economically significant species such as *Apium graveolens* [17], *Lactuca sativa* [18], and *Abelmoschus esculentus* [19]. The findings of all the above studies indicate that smoke can be used to improve growth and crop yields. 

The seed germination process is controlled by many external environmental factors, such as light, temperature, and moisture, as well as by internal growth regulators, such as gibberellins (GAs), abscisic acid (ABA), and the GAs to ABA ratio [20,21,22]. Gibberellin and abscisic acid control seed germination and dormancy initiation by stimulating the synthesis and production of α-amylase during seed germination [23,24]. The role of smoke-water is also important in the production of plant hormones during seed germination. Cembrowska-Lech and Kepczynski [25] reported that smoke-water influenced several biochemical processes, such as the activity of α amylases and β-tublin accumulation in dormant seeds of *Avena fatua* L. In addition, the active compound of smoke-water reduced the ABA level and regulated GA during *Arabidopsis* seed germination [26]. Interestingly, few studies have found evidence that seed dormancy and germination response to smoke are affected by light. Ren et al. [27] reported that germination of lettuce seeds was better promoted under dark conditions by using smoke-water. However, Nelson et al. [28] reported that the Arabidopsis seed germination was observed only under light conditions and not under dark conditions in response to KAR treatment. Therefore, it is important to test the germination in response to smoke-water over a range of light regimes (light and alternating light/darkness) to ensure a proper ecological interpretation of the results [29]. 

Although some previous studies have shown that the smoke-water can be prepared from all plant species, and that their effect is similar on seed germination [30,31], Smith et al. [32] reported that there may be thousands of unknown compounds in smoke, and that the positive effects of smoke on seed germination may depend on the plant species. Additionally, a recent study has reported that the active compounds that can stimulate seed germination have not existed in smoke derived from different legume materials [27]. Also, smoke produced from alfalfa affects seed germination differently compared with that produced from wheat straw and prairie hay [33], indicating that quantitative and qualitative variations exist in smoke solutions derived from different materials. In light of this view, different plant materials have been extensively used to produce smoke-water extracts [34]. These outcomes reflect the fact that the species suitable for producing smoke-water are not fully identified. This would suggest the need for further studies. 

According to the United Nations Food and Agricultural Organization (FAO), tomato and cucumber are among the most important vegetables cultivated and consumed all over the world. Secondly, gladiolus and scotch marigold are very popular ornamental plants in high demand worldwide. Seedling characteristics are considered to be an essential quality aspect affecting plant productivity and are often related to yield. Profitable plant production starts with good quality and healthy seedlings. Seedling quality is a combination of height, diameter, plant nutrition, and root size. Together, these characteristics determine how well the plant will establish itself in the field, and they also affect the rate of survival. 

To the best of our knowledge, most studies on the impact of smoke-water on seed germination have been conducted on the Arabidopsis plant [28,35] and fire-following species [36]. Additionally, most studies investigating the positive effects of smoke on seed germination have been conducted on native species of Australia, South Africa, and California. One of the greatest challenges is that there is little published data on the impact of smoke-water on the seedling vigor and/or plant responses in non-fire-prone environments. Consequently, this study was conducted firstly to evaluate the effects of smoke treatments derived from five plant species on the germination parameters [germination percentage (GP) and germination rate (GR)] of four economically important horticultural crops—cucumber, tomato, gladiolus, and scotch marigold under light and dark conditions, and secondly to assess the impact of the smoke-water derived from these plant species on post-germination parameters and the mineral content of the plants studied. An additional objective was to elucidate the underlying hormonal changes (α-amylase activity and ABA) observed in response to applied seed stimulation treatments. To achieve these goals, smoke samples were collected from burning the soft stems and leaves of white willow, sage, rice straw, rosemary, and lemon eucalyptus, which are common species and can be easily accessed in Egypt.

## 2. Materials and Methods

### 2.1. Plant Materials

The tested plant species included cucumber (*Cucumis sativus* cv. Ishrak) produced by the main vegetable crops and hybrid production project of the Horticulture Research Institute, Agriculture Ministry, Egypt; tomato (*Lycopersicon esculentum* cv. V.385 F1) produced by the Vilmorin company; and gladiolus (*Gladiolus hybrid*) and scotch marigold (*Calendula officinalis*), both of which were freshly collected from the experimental farm of the Faculty of Agriculture, Suez Canal University, Ismailia, Egypt. Seeds and cormels were collected in the summers of 2016 and 2017, and the experiments were performed in the autumns of 2016 and 2017 in laboratory conditions (within one month of collection). Seeds and cormels that appeared viable—based entirely on shape, color, and physical evaluation—were chosen for the experiment.

### 2.2. Production of Plant-Derived Smoke-Water

**Figure 1:**
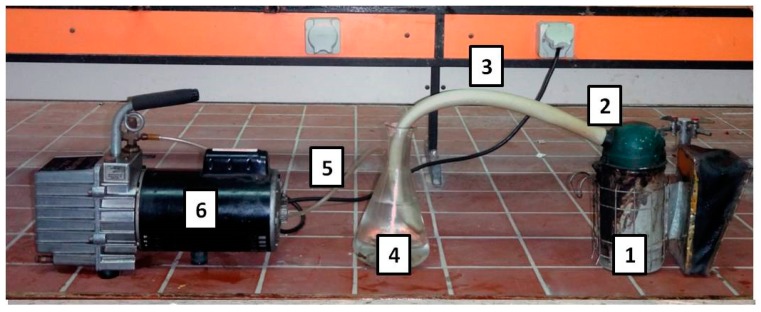
Smoke solution systems. The burning system includes (1) bee smoker, (2) hose clamp, (3) heater hose, (4) conical flask, (5) vacuum tubing, and (6) vacuum. The working framework was constructed and utilized in a fume hood.

Smoke-water was produced by burning 1 kg of dried litter from five plant species—white willow (*Salix alba*), sage (*Salvia officinalis*), rice straw (*Oryza sativa*), rosemary (*Rosmarinus officinalis*), and lemon eucalyptus (*Eucalyptus citriodora*)—in a bee smoker attached by a heater hose to a side-arm flask, as described by Baxter et al. [37] and Coons et al. [38] (see Figure 1). Briefly, the flask was attached to a vacuum water aspirator to bubble smoke through a hose into 1 L of distilled water (DW). The dried plant material was allowed to burn with the smoker open for 30 s [38], after which time the smoker lid was closed, and the tubing was clamped to the opening of the smoker. The smoke was drawn through the water in the flask, dissolving the water-soluble compounds for 30 to 40 min. Bellows on the bee smoker were pumped for the duration of the process to keep the plant material burning and to increase the amount of smoke entering the water in the flask. As the plant material burned, more was added to the bee smoker until the total sample was burned. Once the process of burning the plant material was completed, the system was left to cool completely. After that, the smoke-water solution in the flask was then ready for use in seed treatments. 

### 2.3. First Experiment

The first section of this paper was conducted to examine the influence of smoke-water derived from the five plant species on the germination parameters (GP and GR) under light and dark conditions. The seeds were surface sterilized using a 1:10 (v/v) aqueous solution of NaOCl for 15 min, after which they were washed 3 times with sterile water. In each treatment, four replicates (Petri dishes) of 50 seeds for each species were soaked in smoke-water or DW for 24 h [39]. Six treatments were applied: DW (control), smoke-water from white willow (SW_1_), smoke-water derived from sage (SW_2_), smoke-water derived from rice straw (SW_3_), smoke-water derived from rosemary (SW_4_) and smoke-water derived from lemon eucalyptus (SW_5_). The treated seeds were incubated at a constant temperature (25 °C) under a 16:8 h light/dark photoperiod with a photosynthetic photon flux density of 73 ± 3.5 μmol m^−2^ s^−1^ provided by cool-white fluorescent lamps or under continuous dark treatment in sterile Petri dishes (11-cm diameter) fitted with two sheets of Whatman^®^ No. 1 filter paper and moistened with 5 mL of evenly distributed DW or smoke-water, depending on the treatment. During the experiment, the humidity was checked every day, and water was added as DW or SW (5 mL) when necessary (every 3 days). The total amount of DW or SW added was 35 ml/Petri dish during the entire experiment (21 days).

Germination data were recorded by counting the number of germinated seeds daily for 21 days. The radicle emergence was the criterion used to consider a seed as germinated. After the final count, the GP and GR were calculated using the following formulae [40]:

$$\mathrm{Germination}\;\mathrm{percentage}\;\left(\mathrm{GP}\right)\;=\;\frac{\mathrm{Number}\;\mathrm{of}\;\mathrm{total}\;\mathrm{germinated}\;\mathrm{seeds}}{\mathrm{Total}\;\mathrm{number}\;\mathrm{of}\;\mathrm{seeds}\;\mathrm{tested}}\;\times \;100$$

$$\mathrm{Germination}\;\mathrm{rate}\;\left(\mathrm{GR}\right)\;=\;\frac{\mathrm{Number}\;\mathrm{of}\;\mathrm{germinated}\;\mathrm{seeds}}{\mathrm{Day}\;\mathrm{of}\;\mathrm{first}\;\mathrm{count}}\;+\;\ldots \ldots \;+\;\frac{\mathrm{Number}\;\mathrm{of}\;\mathrm{germinated}\;\mathrm{seeds}}{\mathrm{Day}\;\mathrm{of}\;\mathrm{final}\;\mathrm{count}}$$

#### 2.3.1. α-Amylase Activity

To determine the activity of α-amylase, treated seeds were harvested 24 h after incubation under light and dark conditions, after which they were immediately frozen in liquid N_2_ for subsequent analyses. Then, α-amylase was extracted and assessed according to the method described by Kamran et al. [41] and was expressed as unit per gram (U/g) of seed.

#### 2.3.2. Extraction and Determination of Abscisic Acid Content

The endogenous ABA content in the treated seeds was extracted in accordance with the method described by Qi et al. [42] and was estimated using gas chromatography in accordance with the method of Du and Xu [43] and expressed as nano gram/gram (ng/g) of seed.

### 2.4. Second Experiment

To assess the impacts of the smoke-water derived from the five plant species on seedling growth parameters and mineral content, a second experiment was conducted during two successive seasons (2016 and 2017) in a greenhouse located on a farm of the Faculty of Agriculture, Suez Canal University. The plant seeds and cormels were surface-sterilized as mentioned in the first experiment. In each treatment, the seeds were soaked in SW_1–5_ or DW for 24 h [39]. Subsequently, the treated cucumber, tomato, and scotch marigold seeds, as well as the gladiolus cormels, were sown in plastic trays (50 cells) filled with peat moss and vermiculite (1:1); three trays were used per crop treatment. During the experiment, the trays were irrigated with DW or smoke-water (depending on the treatment) as required. The seedlings were fertilized once a week with a nitrogen-phosphorus-potassium (N-P-K) solution (20-20-20) at a concentration of 1 g/L. After one month, the seedlings were removed and washed with tap water, and then shoot length, root length, and fresh and dry weights of the shoots were measured. The average shoot length, average root length, and GP were evaluated in accordance with the methods of Dhindwal et al. [44] to measure the seedling vigor index as follows:

[SVI = (Average shoot length (cm) + Average root length (cm)) × Germination percentage (%)]

Chlorophyll content in the leaf samples was assessed by a SPAD-502 meter (Minolta Co. Ltd., Osaka, Japan). 

### 2.5. Mineral Nutrition Analysis

Plant samples were collected and subsequently washed under tap water, rinsed with 1% HCl, then thoroughly rinsed three times with distilled water. All samples were dried to constant weight at 70 °C in an oven before being milled to a fine powder with an electric blender and subsequently stored until analysis. For the determination of mineral content, the dried sample powder (0.5 g) was digested in sulfuric acid and hydrogen peroxide as described by Jackson [45]. After the digested materials were diluted, total N was determined using the modified Kjeldahl method. Total P and K were determined in accordance with the AOAC official method [46].

### 2.6. Data Analysis

All statistical procedures with respect to the experimental data were performed using R 3.4.1 software [47]. The analysis of variance was performed with the aov function in the stats R package. Two-way ANOVA was used to analyze data from the first experiment to calculate the main effect of smoke-water and light treatments as well as their interaction on the germination parameters (GP and GR) as well as on α-amylase activity and ABA content. Meanwhile, one-way ANOVA was used to analyze data from the second experiment in a statistical model of completely randomized design to calculate the significant differences between the smoke-water treatments of shoot and root length, fresh and dry weight, chlorophyll content, seedling vigor index, and mineral composition (N, P, and K). Significance levels were calculated at the 0.01 level using Duncan’s multiple range tests function as implemented in the agricolae R package [47]. Since there were no statistically significant differences in the studied parameters between the two seasons, the average of the two seasons is discussed herein. The statistical analysis of percentage germination was performed on arcsine-transformed data.

## 3. Results

### 3.1. First Experiment: Effects of Smoke-Water and Light versus Darkness on Germination Parameters, α-Amylase Activity, and Abscisic Acid Content

Analysis of variance showed that smoke-water treatment, light condition, and their interaction had significant effects on GP, GR, α-amylase activity, and ABA content in tomato, gladiolus, and scotch marigold. However, in cucumber, significant effects of smoke-water treatment and significant smoke-water-light interaction were observed for all studied traits, whereas there was no significant difference between the two light conditions in terms of GP, GR, and α-amylase activity. The data presented in Table 1 show the main effect of smoke-water treatments on GP, GR, α-amylase activity, and abscisic acid content in seeds and cormels of the studied plant species treated with five different smoke-water treatments and DW control. It shows that there were significant differences among smoke-water treatments in terms of GP and GR, α-amylase activity, and abscisic acid content in all studied crops. In addition, Table 1 shows that, compared to the control and other smoke-water treatments, both SW_1_ and SW_5_ treatments recorded the highest values of GP, GR, and α-amylase activity. However, they recorded the lowest values of abscisic acid content in all studied crops in the light and the dark conditions. 

**Figure 2:**
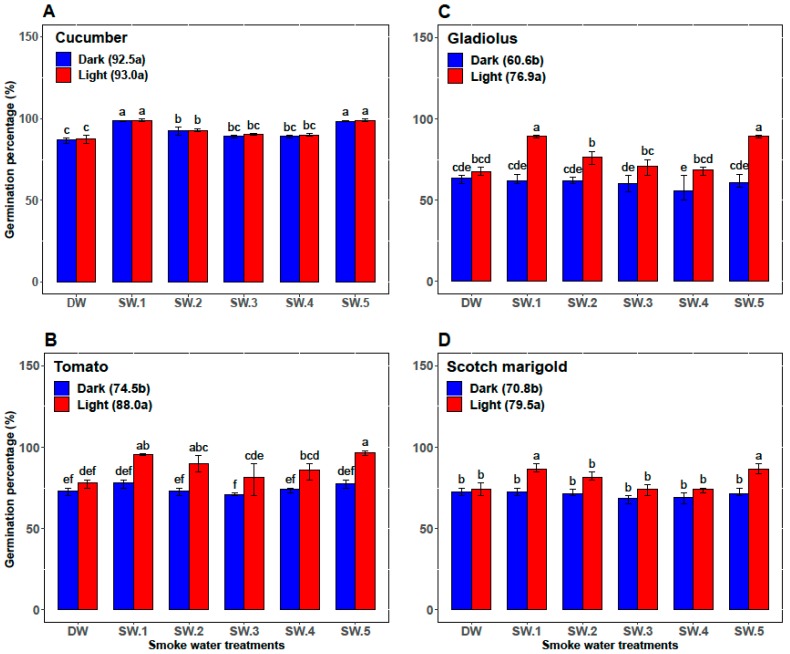
Comparative effect of different smoke-waters on the germination percentage of cucumber (**A**), tomato (**B**), gladiolus (**C**), and scotch marigold (**D**) under light and dark conditions. Bars within a panel with different letters are significantly different (*p* < 0.01) based on Duncan.

**Figure 3:**
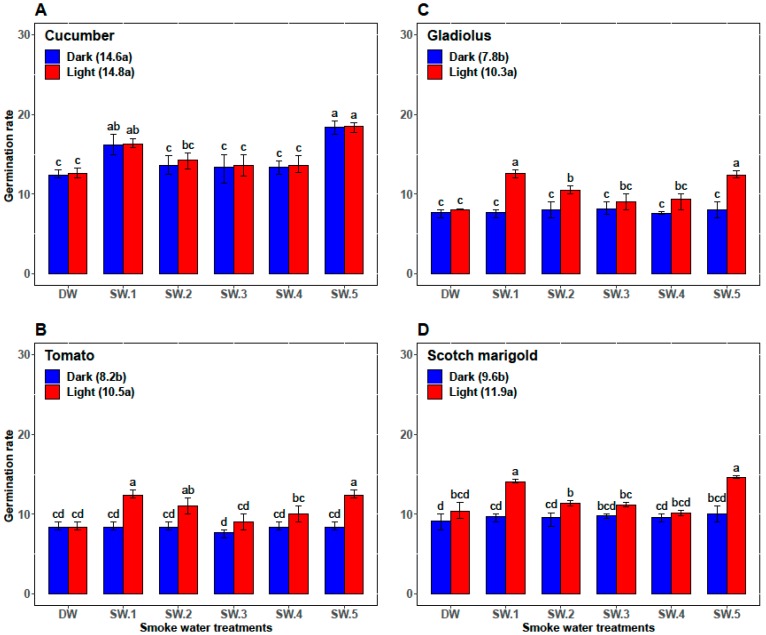
Comparative effect of different smoke-waters on the germination rate of cucumber (**A**), tomato (**B**), gladiolus (**C**), and scotch marigold (**D**) under light and dark conditions. Bars within a panel with different letters are significantly different (*p* < 0.01) based on Duncan.

**Figure 4:**
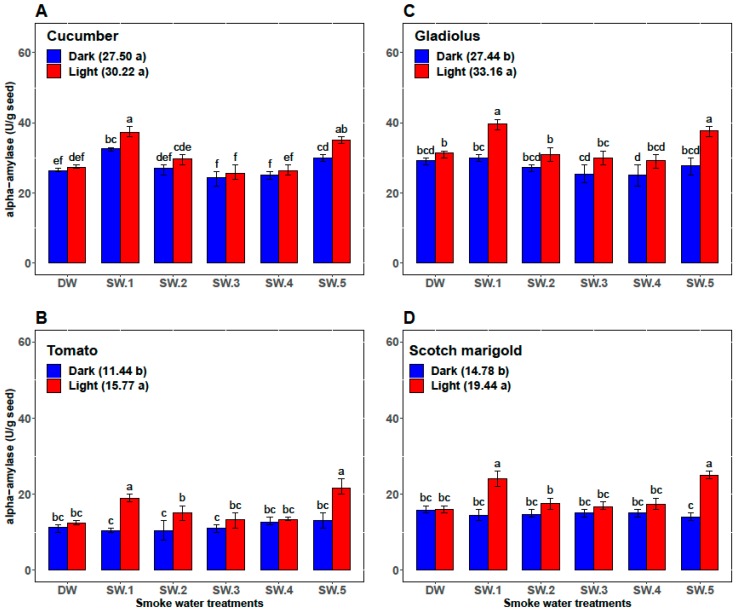
The effects of smoke-water treatments on α-amylase activity in seeds of cucumber (**A**), tomato (**B**), gladiolus (**C**), and scotch marigold (**D**) under light and dark conditions. Bars within a panel with different letters are significantly different (*p* < 0.01) based on Duncan.

**Figure 5:**
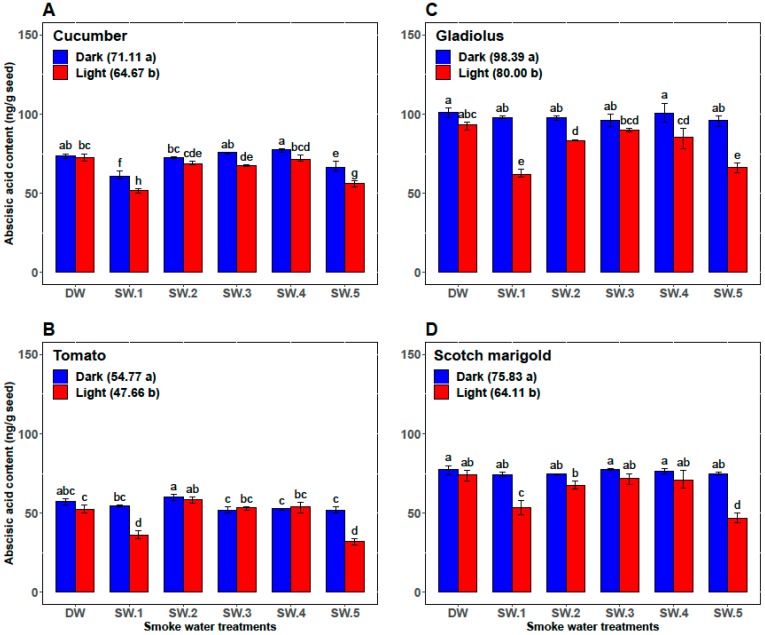
The effects of smoke-water treatments on abscisic acid content in seeds of cucumber (**A**), tomato (**B**), gladiolus (**C**), and scotch marigold (**D**) under light and dark conditions. Bars within a panel with different letters are significantly different (*p* < 0.01) based on Duncan.

In cucumber, the GP, the GR, and the α-amylase activity in smoke-water treatments under the light conditions (93.0%, 14.8, and 30.2, respectively) were overall not significantly higher than those under dark conditions (92.5%, 14.6, and 27.5, respectively). However, abscisic acid content was significantly higher (71.1) under dark conditions than under light conditions (64.7). Among all the smoke-water treatments, the highest values of GP and GR were achieved in cucumber seed treated by SW_1_ and SW_5_ under light and/or dark conditions (Figure 2A and Figure 3A). However, the cucumber seeds treated with SW_1_ and SW_5_ recorded the highest values of α-amylase activity and the lowest values of abscisic acid content under light conditions (Figure 4A and Figure 5A).

Compared with darkness, light significantly promoted GP, GR, and α-amylase activity of tomato seeds (74.5%, 8.2, and 11.44, respectively, versus 88.0%, 10.5, and 15.77, respectively) and significantly decreased the abscisic acid content (54.77 versus 47.66) over the control and all smoke-water treatments. Also, both SW_1_ and SW_5_ gave the highest GP, GR, and α-amylase and the lowest abscisic acid content in tomato seeds under only light conditions (Figure 2B, Figure 3B, Figure 4B, and Figure 5B).

In gladiolus, light conditions successfully broke the dormancy of the gladiolus cormels and improved their GP, GR, and α-amylase activity (60.60%, 7.80, and 27.44, respectively, versus 76.90%, 10.30, and 33.16, respectively) and reduced their abscisic acid content (98.39 versus 80.00) over all smoke-water treatments. Regarding the interaction between the smoke-water treatments and light conditions, both treatments using SW_1_ and SW_5_ recorded the highest values of GP, GR, and α-amylase and the lowest abscisic acid content under light conditions (Figure 2C, Figure 3C, Figure 4C, and Figure 5C). 

Additionally, light promoted the GP, GR, and α-amylase activity (79.5%, 11.9, and 19.44, respectively) compared with dark conditions (70.8%, 9.6, and 14.78, respectively) and reduced the abscisic acid content in treated seeds of scotch marigold by 18.28%. Among the 12 treatments, the combinations of SW_1_ and SW_5_ under light conditions achieved the highest values of GP, GR, and α-amylase and the lowest abscisic acid content in the treated scotch marigold seeds (Figure 2D, Figure 3D, Figure 4D, and Figure 5D).

### 3.2. Second Experiment: Effects of Smoke-Water on Growth Parameters and Macroelement Contents

Table 2 shows that there were highly significant differences in growth parameters among the smoke-water treatments in all the studied crops with the exception of the chlorophyll content in cucumber. Overall, exposure to SW_1_ and SW_5_ followed by SW_2_ had the most stimulating effect on growth parameters in all studied crops (Table 2). With respect to the mineral content, not all smoke-water treatments resulted in N, P, and K levels that were significantly higher than those in the control treatment. SW_1_ and SW_5_ treatments significantly increased the levels of N and P in all the studied crops; however, they resulted in a significant increase in the level of K only in gladiolus (Table 2).

## 4. Discussion

Seed germination and the production of healthy seedlings are important principles for improving the productivity of all horticultural crops. Plant-derived smoke has been recognized as a promising stimulator for germination. In this study, we chose smoke-waters that are easily obtainable and a technique that is convenient and inexpensive for farmers. The current study clearly shows that plant-derived smoke-water treatments, particularly the smoke-water derived from white willow (SW_1_) and lemon eucalyptus (SW_5_), effectively improved GP, GR, and seedling growth of the tested crops. In accordance with our results, previous studies have shown that smoke-water and smoke-derived compounds have a positive effect on seed germination and the post-germination parameters of crops such as wheat [47], onion [48], tomato [49], okra [50], papaya [51], and lettuce [14,27]. Additionally, our results provide evidence that smoke-water can actively break dormancy in gladiolus [52]. Several explanations have been given to understand the mechanism of how smoke-water can affect seed germination. Some scientists have suggested that smoke-water can increase the efficiency of an embryo’s oxygen absorption and water uptake [53]. However, the majority of scientists show that plant-derived smoke contains several primitive compounds, such as cyanohydrins and hydrocarbons, that stimulate seed germination [12,14,22,54,55]. They reported that the smoke-water compounds were detected and confirmed by chemical analysis, and these compounds could affect seed germination by regulating the biosynthesis and signaling of several plant hormones involved in the seed germination process, such as ABA, GA, and auxin. 

Phytohormones play a dominant role in controlling seed germination [20,22,56]. GA and ABA contents and their ratio play a crucial role in seed germination [57,58]. Briefly, GA plays a key role in promoting germination, whereas ABA has an inhibitory effect [59]. Active compounds of smoke (KARs) may play a role in regulating seed germination by interacting with endogenous phytohormone signaling [14,22]. In the present study, ABA quantification revealed that SW_1_ and SW_5_ treatments convincingly reduced the ABA contents during the seed germination of cucumber, tomato, and scotch marigold, as well as the germination of gladiolus cormels. These results reflect those of Nelson et al. [54], who also found that KARs reduced ABA levels and regulated GA during *Arabidopsis* seedling germination. 

In addition to phytohormones, the content of starch is an exceptional fundamental factor in seed germination. Starch breakdown is responsible for the release of carbohydrates and their subsequent use, mainly for sucrose synthesis and respiration [60]. Several enzymes take part in the breakdown of starch. However, the enzyme most frequently credited with the initial attack on starch granules is α-amylase [61]. In contrast to ABA content, α-amylase activity was highest in all crops treated with SW_1_ and SW_5_. In accordance with the present results, Karman et al. [41] found that plant-derived smoke solution treatments significantly increased the α-amylase activity and reduced the ABA contents in barnyard grass. Also, smoke-water accelerated α-amylase activity and reduced starch content in wild oat seeds [25]. One of the clearest findings of the present study was the relationships between α-amylase activity, ABA content, and germination parameters in all studied crops. Clearly, SW_1_ and SW_5_ significantly improved the GP and GR by increasing the activity of α-amylase and reducing the ABA level in all studied crops under light conditions. 

Interestingly, the stimulating effects of the smoke-water treatments, particularly the smoke-water derived from white willow (SW_1_) and lemon eucalyptus (SW_5_), on ABA and α-amylase content as well as the percentage and rate of germination occurred only under light conditions in tomato, gladiolus, and scotch marigold. However, they were absent or weak under dark conditions. Contrarily, the stimulatory effects caused by the smoke-water treatments were observed under light and dark conditions in cucumber. According to these data, we can infer that smoke-water plays an important role in stimulating seed germination by regulating ABA and α-amylase activities, and its effect becomes more pronounced under light conditions. However, this stimulating effect of smoke-water can be masked under dark conditions. Indeed, there is scientific debate about the effects of smoke-water and its active compounds (KARs) on germination parameters and whether smoke-water can substitute for light conditions or not. The germination percentage of some light sensitive crops such as lettuce seeds (*Lactuca sativa*) can be significantly increased under dark conditions by using smoke-water [6,27]. On the other hand, Nelson et al. [28] showed that the effect of KAR on *Arabidopsis* seed germination was observed only under light conditions and not under dark conditions, which confirms the results found in tomato, gladiolus, and scotch marigold in the current study. In contrast to this promotion effect, Meng et al. [58] reported that KAR has no effect on soybean seed germination under light or dark conditions, but a negative impact occurred under shaded conditions. A comparison of our findings with those of other studies confirms that smoke-water has distinct biological functions with regard to seed germination. Nevertheless, its influence may be affected by the presence/absence of light and the plant species [6,27,54].

Jäger et al. [30] assumed that all plant materials are suitable for the smoke-water preparation. However, in contrast with these expectations, the results of the current study convincingly indicate that the stimulating effects of smoke-water on the germination and post germination parameters of cucumber, tomato, gladiolus, and scotch marigold varied in response to the different smoke-water types. Different responses among the five derived smoke-water treatments indicate that smoke originating from different plant materials may not contain either the same promotive compounds or may contain distinctive compounds. In agreement with our results, Ren et al. [27] as well as Ren and Bai [33] reported different germination effects in response to treatment with smoke-water derived from alfalfa (*Medicago sativa*), wheat straw (*Triticum aestivum*), and prairie hay (*Festucahallii*), and concluded that primary components such as KAR1 do not exist in the smoke-water derived from all plant materials; those authors also concluded that the smoke solutions obtained from different plant materials contain several active components that are qualitatively and quantitatively different, which in turn may explain the different effects of smoke-water treatments on seed germination and post-germination parameters in the current study.

In contrast to the promoting effect of SW_1_ and SW_5_, both SW_3_ and SW_4_ treatments achieved a neutral effect compared to the control on germination and post-germination parameters. This neutral effect of these treatments could have been due to one of the following explanations: (1) the absence of the active compound, mainly KARs, (2) the existence of inhibitor compounds, or (3) the high concentration of the active compounds in the smoke produced by burning rice straw and rosemary (SW_3_ and SW_4_, respectively). In this regard, Ren et al. [27] and Ren and Bai [33] reported that KARs do not exist universally in smoke from all plant materials, as mentioned above. Also, the existence of some inhibitor compounds in the smoke, such as butenolide 3.4.5-trimethylfuran-2 (5H), that could inhibit germination and significantly reduce the effect of KARs was reported [13]. In addition, it was reported that low concentrations of smoke-water significantly improved germination activity compared to high concentrations [51]. Therefore, further studies are needed to explore the compounds existing in the smoke and to investigate the dilution effect of smoke from these plant materials on germination and post-germination parameters. 

**Figure 6:**
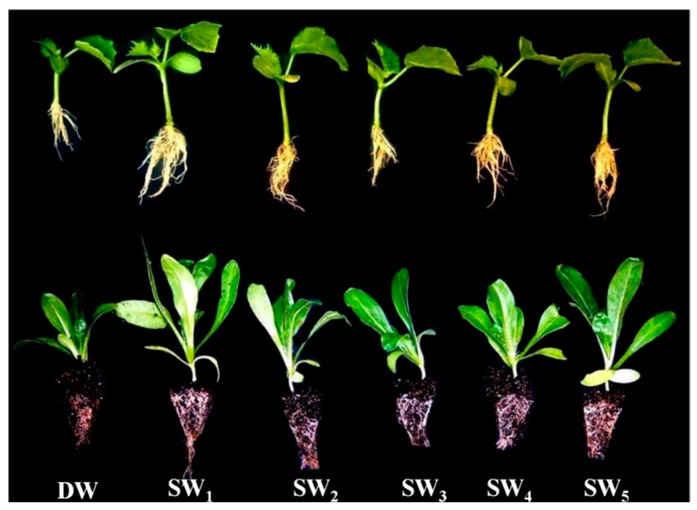
Effect of DW and smoke-water (SW_1–5_) on seedling growth of cucumber (up) and scotch marigold (down).

Another important finding of the present study was that, compared to the control and other smoke-water treatments, SW_1_ and SW_5_ treatments led to a significant increase in post-germination parameters such as shoot length, root length, fresh and dry weights, chlorophyll content, and SVI. These findings were consistent with those in the study conducted by Sparg et al. [62] and Zhou et al. [63], who reported that seedlings grown from seeds treated with plant-derived smoke have longer roots and shoots as well as higher fresh and dry biomass than the control plants. In addition, treatment with SW_1_ or SW_5_ significantly and consistently increased the levels of N and P in the shoot of the four studied crops whilst significantly increasing the levels of K only in gladiolus. These results were in agreement with recent studies in which smoke-water promoted several growth attributes of papaya seedlings by improving the mineral nutrient content [51]. This result indicates that smoke-water treatments have a positive effect in improving nutrient uptake, translocation, and utilization efficiency, leading to healthy seedlings with a significant reduction in the amount of fertilizer required and consequently the costs. This indicates that smoke solutions can enhance the uptake of nutrients by promoting the root system of the studied plants, as seen in Figure 6 [48]. It also points out that the smoke-water approach is a promising alternative to seed priming approaches such as osmopriming, hormonal priming, hydropriming, and matrix priming, which are used to improve seed germination, as well as to plant growth and development in several plant species, since it is a cost-effective, easy to produce, and practical method for farmers to obtain healthy and uniform seedlings under normal or unfavorable conditions.

## 5. Conclusions

The most obvious finding to emerge from this study is that not all plants are suitable for the preparation of smoke-water. The smoke-water derived from white willow and lemon eucalyptus positively affected both the germination and the growth parameters of cucumber, tomato, gladiolus, and scotch marigold by enhancing the activity of the α-amylase enzyme and mineral uptake. However, this stimulatory effect of smoke-water from white willow and lemon eucalyptus was masked under dark conditions in all studied crops with the exception of cucumber. Both the lower costs for producing smoke-water and its technical simplicity make smoke-water a useful and practical tool in good agricultural practice for improving seed germination and growth during seedling production within nurseries. More broadly, it will be interesting to highlight the potential effects of smoke-water derived from white willow and lemon eucalyptus on different pyrolysis temperatures as well as different dilutions of smoke-water derived from these two species on seedling production and the alleviation of abiotic stress, such as that caused by drought and salinity in some horticultural crops.

## Figures and Tables

**Table 1:** The main effect of smoke-water (SW) treatments on germination percentage (GP), germination rate (GR), α-amylase activity, and abscisic acid (ABA) content in the studied crops.

Plant	Smoke-Water	Germination Percentage (%)	Germination Rate	α-Amylase (U/g Seed)	Abscisic Acid (ng/g Seed)
Cucumber	DW	87.33 ± 1.97 c	12.47 ± 0.57 c	26.83 ± 0.75 cd	73.00 ± 2.00 ab
	SW_1_	98.67 ± 0.82 a	16.28 ± 0.88 b	35.00 ± 2.76 a	56.33 ± 5.47 d
	SW_2_	92.67 ± 1.75 b	13.48 ± 1.42 c	28.33 ± 2.07 c	70.67 ± 2.34 b
	SW_3_	89.83 ± 0.98 c	13.97 ± 1.06 c	25.00 ± 2.00 d	71.67 ± 4.41 ab
	SW_4_	89.67 ± 1.03 c	13.53 ± 0.87 c	25.67 ± 1.37 d	74.50 ± 3.45 a
	SW_5_	98.5 ± 0.84 a	18.46 ± 0.70 a	32.50 ± 2.88 b	61.17 ± 6.15 c
Tomato	DW	75.83 ± 3.76 b	8.33 ± 0.52 b	11.83 ± 0.98 b	54.83 ± 3.43 b
	SW_1_	86.83 ± 9.50 a	10.33 ± 2.25 a	14.67 ± 4.80 ab	45.33 ± 10.37 c
	SW_2_	81.67 ± 9.83 ab	9.67 ± 1.63 ab	12.67 ± 2.80 b	59.17 ± 2.04 a
	SW_3_	76.17 ± 8.95 b	8.33 ± 1.03 b	12.17 ± 1.94 b	52.67 ± 1.63 b
	SW_4_	80.00 ± 7.46 ab	9.17 ± 1.17 ab	13.00 ± 0.89 b	53.33 ± 2.42 b
	SW_5_	87.00 ± 10.75 a	10.33 ± 2.25 a	17.33 ± 5.09 a	42.00 ± 11.10 c
Gladiolus	DW	65.33 ± 3.27 bc	7.87 ± 0.43 c	30.33 ± 1.51 bc	97.17 ± 5.23 a
	SW_1_	75.67 ± 15.15 a	10.12 ± 2.73 a	34.83 ± 5.42 a	80.00 ± 19.80 c
	SW_2_	69.33 ± 8.55 ab	9.25 ± 1.54 ab	29.17 ± 2.71 cd	90.50 ± 07.92 b
	SW_3_	65.33 ± 7.39 bc	8.58 ± 0.92 bc	27.67 ± 2.8 cd	93.17 ± 4.36 ab
	SW_4_	62.00 ± 8.83 c	8.47 ± 1.20 bc	27.17 ± 3.31 d	93.00 ± 10.14 ab
	SW_5_	75.00 ± 15.99 a	10.20 ± 2.51 a	32.67 ± 5.79 ab	81.33 ± 16.72 c
Scotch marigold	DW	73.33 ± 3.20 bc	9.77 ± 1.13 b	15.83 ± 0.98 c	75.67 ± 3.50 a
	SW_1_	79.50 ± 8.22 a	11.88 ± 2.46 a	19.17 ± 5.53 ab	64.00 ± 11.71 b
	SW_2_	76.50 ± 6.12 ab	10.46 ± 1.16 b	16.17 ± 2.04 bc	71.17 ± 4.17 a
	SW_3_	71.17 ± 4.26 c	10.49 ± 0.83 b	15.83 ± 1.33 c	74.67 ± 3.72 a
	SW_4_	71.50 ± 3.73 c	9.85 ± 0.49 b	16.17 ± 1.72 bc	73.50 ± 4.85 b
	SW_5_	79.17 ± 8.64 a	12.32 ± 2.62 a	19.50 ± 6.09 a	60.83 ± 15.65 b

Values with the same letter in each column are not significantly different according to Duncan’s difference test (*p* ≤ 0.01). The values presented in this table are the averages ± standard deviation under light and dark conditions together for each plant species. *DW = distilled water.

**Table 2:** Effect of different smoke-waters on growth parameters and macro-elements content in cucumber, tomato, gladiolus, and scotch marigold. The data represented are the average ± standard deviation.

Plant	Smoke-Water	Shoot Length (cm)	Root Length (cm)	Fresh Weight (gm)	Dry Weight (gm)	Chlorophyll Content	Seedling Vigor Index	N (%)	P (%)	K (%)
Cucumber	DW	7.67 ± 0.58 b	5.33 ± 0.62 b	2.07 ± 0.41 b	0.12 ± 0.03 d	63.00 ± 1.53 a	1170.00 ± 90.00 c	1.35 ± 0.15 c	0.16 ± 0.02 c	2.07 ± 0.12 a
	SW_1_	10.00 ± 1.00 a	7.67 ± 0.58 a	3.15 ± 0.38 a	0.33 ± 0.05 a	71.00 ± 1.50 a	1702.00 ± 66.36 a	2.43 ± 0.18 ab	0.24 ± 0.08 ab	2.27 ± 0.25 a
	SW_2_	9.00 ± 1.00 ab	7.00 ± 1.00 a	2.98 ± 0.45 ab	0.22 ± 0.03 bc	67.33 ± 1.00 a	1487.00 ± 146.65 ab	2.1 0 ± 0.10 b	0.20 ± 0.02 bc	2.23 ± 0.32 a
	SW_3_	8.00 ± 1.00 ab	5.67 ± 0.58 b	2.45 ± 0.23 ab	0.19 ± 0.01 cd	64.33 ± 1.53 a	1252.00 ± 132.18 bc	1.67 ± 0.15 c	0.17 ± 0.05 c	2.17 ± 0.25 a
	SW_4_	7.68 ± 0.58 b	5.35 ± 0.58 b	2.88 ± 0.38 ab	0.18 ± 0.03 cd	63.67 ± 1.00 a	1196.00 ± 113.25 bc	1.47 ± 0.12 c	0.18 ± 0.02 c	2.10 ± 0.36 a
	SW_5_	9.67 ± 0.59 ab	8.00 ± 1.00 a	3.20 ± 0.28 a	0.28 ± 0.03 ab	72.00 ± 0.58 a	1684.67 ± 153.58 a	2.47 ± 0.10 a	0.27 ± 0.02 a	2.24 ± 0.12 a
Tomato	DW	11.00 ± 0.58 b	7.67 ± 1.00 b	1.68 ± 0.20 c	0.16 ± 0.02 b	60.67 ± 3.12 b	1512.67 ± 109.12 c	2.24 ± 0.25 b	0.23 ± 0.03 b	3.78 ± 0.29 a
	SW_1_	14.33 ± 1.15 a	9.60 ± 0.53 ab	2.57 ± 0.12 a	0.23 ± 0.04 a	75.67 ± 2.35 a	2194.13 ± 99.68 a	3.20 ± 0.3 a	0.35 ± 0.01 a	4.42 ± 0.14 a
	SW_2_	13.00 ± 1.00 ab	8.43 ± 1.52 b	2.35 ± 0.13 ab	0.17 ± 0.01 b	63.67 ± 3.09 b	1840.00 ± 83.25 b	2.77 ± 0.35 ab	0.33 ± 0.02 a	4.18 ± 0.50 a
	SW_3_	11.33 ± 1.15 b	8.00 ± 1.85 b	1.78 ± 0.09 c	0.16 ± 0.02 b	61.67 ± 5.29 b	1517.00 ± 159.12 c	2.43 ± 0.25 b	0.28 ± 0.03 ab	4.00 ± 0.20 a
	SW_4_	11.67 ± 1.01 b	8.83 ± 1.22 b	1.87 ± 0.19 bc	0.16 ± 0.04 b	62.83 ± 7.00 b	1658.17 ± 92.35 bc	2.45 ± 0.31 b	0.25 ± 0.05 b	4.17 ± 0.26 a
	SW_5_	15.00 ± 1.00 a	11.00 ± 0.76 a	2.45 ± 0.41 a	0.26 ± 0.01 a	74.33 ± 5.13 a	2423.33 ± 126.23 a	3.25 ± 0.15 a	0.34 ± 0.02 a	4.33 ± 0.42 a
Gladiolus	DW	13.83 ± 1.00 b	6.67 ± 1.00 b	1.13 ± 0.12 b	0.16 ± 0.02 b	32.33 ± 5.52 b	1335.83 ± 155.45 c	1.86 ± 0.15 b	0.31 ± 0.03 b	3.03 ± 0.13 b
	SW_1_	17.00 ± 1.50 a	9.00 ± 0.76 a	2.40 ± 0.15 a	0.24 ± 0.05 a	49.33 ± 4.04 a	2210.00 ± 123.58 a	3.05 ± 0.24 a	0.44 ± 0.02 a	4.21 ± 0.05 a
	SW_2_	15.67 ± 1.25 ab	8.87 ± 1.76 a	1.40 ± 0.10 b	0.19 ± 0.01 ab	37.00 ± 2.65 b	1689.00 ± 128.36 b	2.17 ± 0.29 b	0.34 ± 0.04 b	3.38 ± 0.33 b
	SW_3_	15.00 ± 1.47 ab	7.97 ± 1.58 ab	1.30 ± 0.17 b	0.16 ± 0.01 b	35.33 ± 1.15 b	1489.33 ± 155.25 c	1.94 ± 0.08 b	0.32 ± 0.03 b	3.14 ± 0.22 b
	SW_4_	14.33 ± 1.22 ab	8.47 ± 1.47 ab	1.17 ± 0.15 b	0.19 ± 0.05 ab	34.67 ± 5.03 b	1444.50 ± 106.37 c	1.92 ± 0.14 b	0.33 ± 0.02 b	3.15 ± 0.18 b
	SW_5_	16.73 ± 0.47a	9.17 ± 1.50 a	2.47 ± 0.11 a	0.20 ± 0.01 ab	50.67 ± 5.59 a	2272.33 ± 136.35 a	2.90 ± 0.27 a	0.37 ± 0.02 ab	3.97 ± 0.16 a
Scotch marigold	DW	8.83 ± 1.26 c	7.37 ± 1.58 b	3.27 ± 1.10 c	0.28 ± 0.04 b	42.33 ± 4.16 b	1160.83 ± 205.31 c	1.49 ± 0.08 b	0.25 ± 0.03 b	2.03 ± 0.16 a
	SW_1_	13.40 ± 1.22 a	10.50 ± 1.50 a	5.93 ± 0.42 a	0.60 ± 0.09 a	56.6 ± 3.54 a	1948.67 ± 169.18 a	2.03 ± 0.06 a	0.35 ± 0.09 ab	2.15 ± 0.20 a
	SW_2_	11.23 ± 1.25 abc	9.00 ± 1.23 ab	4.66 ± 1.01 b	0.56 ± 0.06 a	45.33 ± 4.35 b	1578.97 ± 119.18 b	1.70 ± 0.10 b	0.27 ± 0.06 ab	2.08 ± 0.17 a
	SW_3_	10.5 ± 1.50 bc	8.77 ± 1.25 b	3.40 ± 0.61 c	0.32 ± 0.05 b	43.33 ± 5.02 b	1343.33 ± 81.23 bc	1.55 ± 0.09 b	0.26 ± 0.05 b	2.06 ± 0.13 a
	SW_4_	10.17 ± 1.76 bc	7.67 ± 1.10 b	3.70 ± 0.85 bc	0.31 ± 0.02 b	42.67 ± 3.25 b	1227.33 ± 162.74 c	1.64 ± 0.06 b	0.25 ± 0.05 b	2.03 ± 0.23 a
	SW_5_	12.17 ± 1.29 ab	10.5 ± 1.50 a	6.20 ± 0.21 a	0.60 ± 0.02 a	54.67 ± 2.12 a	1851.67 ± 89.28 a	2.06 ± 0.12 a	0.37 ± 0.03 a	2.12 ± 0.17 a

Values with the same letter in each column are not significantly different according to Duncan’s difference test (*p* ≤ 0.01).
